# Impact of COVID-19 on mortality in out-of-hospital cardiac arrest patients with return of spontaneous circulation: a retrospective cohort study

**DOI:** 10.1186/s13049-025-01395-2

**Published:** 2025-05-01

**Authors:** Nai-Chen Shih, Han-Wei Yeh, Shun-Fa Yang, Yu-Hsun Wang, Chung-Hsien Chaou, Chao-Bin Yeh

**Affiliations:** 1 Institute of Medicine, Chung Shan Medical University, Taichung, Taiwan; 2 Department of Family Medicine, Taichung Veterans General Hospital, Taichung, Taiwan; 3 Department of Emergency Medicine, Linkou Chang Gung Memorial Hospital, Taoyuan City, Taiwan; 4 School of Medicine, Chang Gung University, Guishan, Taoyuan City, Taiwan; 5 Chang-Gung Medical Education Research Centre, Chang-Gung Memorial Hospital, Taoyuan City, Taiwan; 6 Department of Medical Research, Chung Shan Medical University Hospital, Taichung, Taiwan; 7 Department of Emergency Medicine, School of Medicine, Chung Shan Medical University, No.110, Sec. 1, Jianguo N. Rd., Taichung, 402 Taiwan; 8 Department of Emergency Medicine, Chung Shan Medical University Hospital, Taichung, Taiwan

**Keywords:** COVID-19 infection, Out-of-hospital cardiac arrest, Mortality, Retrospective cohort study

## Abstract

**Background:**

This study aims to compare the mortality rates of OHCA patients with and without COVID-19 infection across different follow-up periods and explores the factors may play a significant role in determining OHCA outcomes.

**Methods:**

This study utilized data from the US Collaborative Network in TriNetX. A total of 25,271 hospitalized OHCA patients were recruited from records spanning from January 1, 2020, to December 31, 2023. Study population divided into two groups, COVID-19 positive and COVID-19 negative groups. The mortality risk of the two groups was observed based on different follow-up periods. Subgroup analyses on sex, age, antivirals use, COVID-19 virus variant epidemic period were also conducted.

**Results:**

Our study included 2,776 patients in each group (COVID vs. non-COVID). The primary outcome was mortality at 14-day and 90-day follow-ups. COVID-19 patients had a lower 14-day mortality (HR 0.82, 95% CI: 0.76–0.88) but higher 90-day mortality (HR 1.16, 95% CI: 1.09–1.24) compared to non-COVID-19 patients. Secondary outcomes included higher mortality in COVID-19 patients under 65, and this trend persisted in those aged 65 and over. Male COVID-19 patients had elevated mortality risk. The Alpha and Delta variant period showed a higher mortality rate for COVID-19 patients than non-COVID-19 patients.

**Conclusion:**

COVID-19 was associated with a higher risk of mortality in OHCA patients.

**Supplementary Information:**

The online version contains supplementary material available at 10.1186/s13049-025-01395-2.

## Background

During the severe acute respiratory syndrome coronavirus-2 (SARS-CoV-2) pandemic, the incidence and mortality rates of out-of-hospital cardiac arrest (OHCA) significantly increased compared with the pre-pandemic levels [1, 2]. The chain of survival for OHCA patients is critical, consisting of early activation of emergency response services, immediate high-quality CPR, early defibrillation, early advanced cardiac life support, and advanced post-cardiac arrest care [3]. The SARS-CoV-2 pandemic potentially disrupted various stages of the chain of survival for OHCA patients, including leading to delays in emergency medical services (EMS) response times and a rise in advanced airway management by EMS [4, 5]. These disruptions extend beyond the prehospital setting. Pandemic-related health system issues, such as overwhelmed emergency medical services and postponed consultations, likely also played a role in the effect of the SARS-CoV-2 pandemic on OHCA care and deaths [6].

While numerous studies have explored the impact of the SARS-CoV-2 pandemic on OHCA incidence, resuscitation rates, and mortality, few investigations have specifically examined outcomes among OHCA patients diagnosed with COVID-19 infection [1, 7]. A study by Sultanian et al. revealed that COVID-19-positive patients experienced the most significant decline in survival, with 83.4% mortality within 24 h [8]. Moreover, compared to COVID-19-negative patients, COVID-19-positive patients have a 3.4-fold higher risk of 30-day mortality [8]. In addition to older age and comorbidities, secondary bacterial infection was a significant contributor to mortality among COVID-19-positive patients, compared to those who are COVID-19-negative [9].

To address the limitations of previous studies that primarily focused on the COVID-19 pandemic rather than patients diagnosed with COVID-19 infection, this study specifically compared the mortality rate of OHCA among COVID-19 patients. In addition to comparing mortality risks among OHCA patients with and without COVID-19 across different follow-up periods, we also conducted subgroup analyses based on various demographic factors, including age, gender, antiviral medication use, and COVID-19 virus variant epidemic period.

## Methods

### Study design and data sources

This retrospective cohort analysis utilized the TriNetX analytics platform, a web-based database housing deidentified electronic health records (EHRs) from over 250 million patients across various countries. The database encompasses a wide array of data, including vital demographics, diagnoses (utilizing ICD-10-CM codes), medications (categorized by RxNorm, Anatomical Therapeutic Chemical codes, or Veterans Affairs Drug Classification system), procedures (classified by ICD-9-PCS, Current Procedural Terminology, Systematic Nomenclature of Medicine, or Healthcare Common Procedure Coding System), and laboratory measurements (identified by Logical Observation Identifiers Names and Codes). The use of deidentified data in this retrospective study was exempt from Institutional Review Board (IRB) approval. The analysis focused on a cohort from the USA collaborative network within the TriNetX database, which comprises around 110 million patients.

### Study participants

**Fig. 1:**
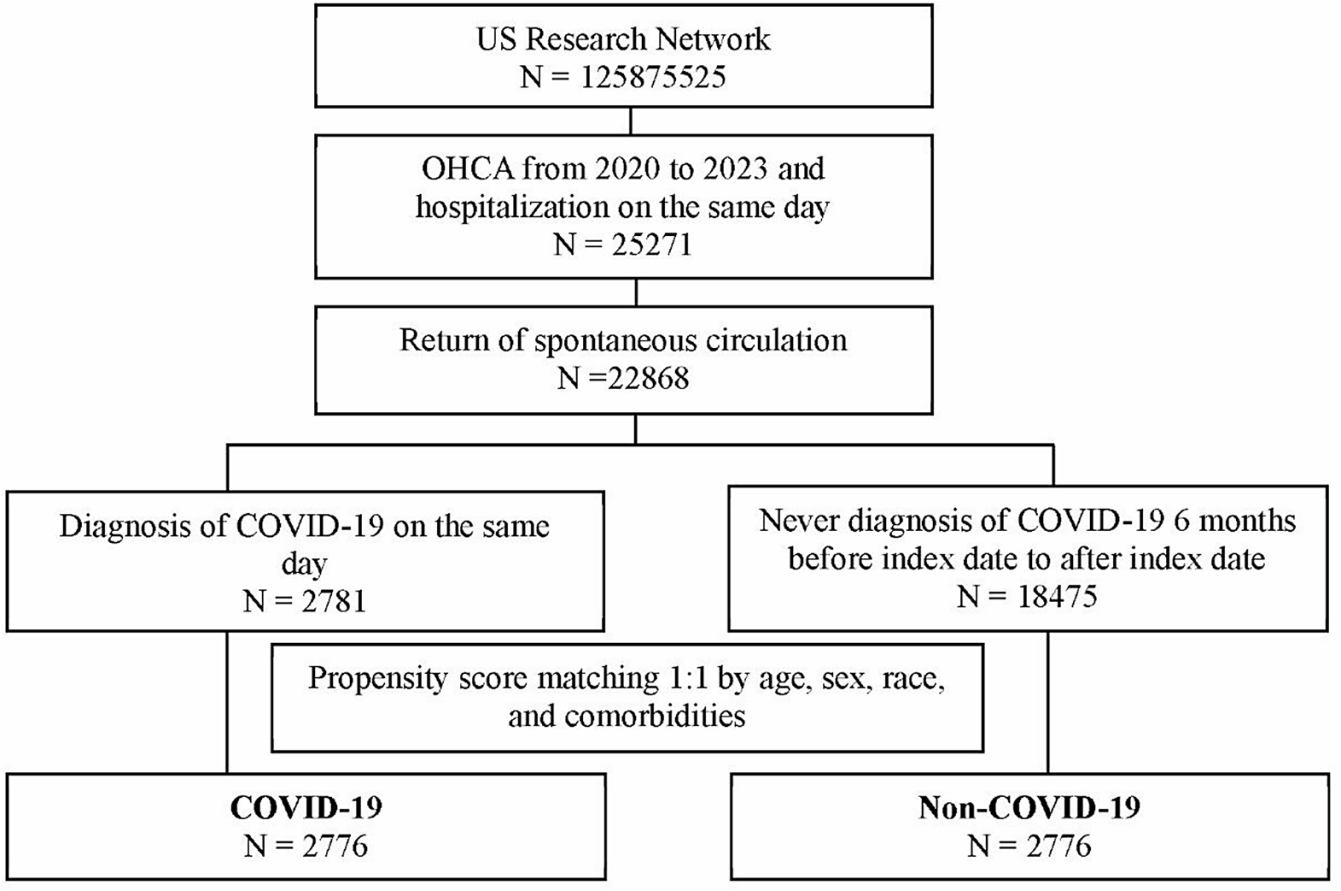
Flowchart illustrating participants’ selection

Figure 1 illustrates the cohort construction flow chart. The study group comprised individuals who experienced an out-of-hospital cardiac arrest (OHCA) and were subsequently hospitalized between 2020 and 2023. OHCA was identified during emergency visits with a diagnosis of cardiac arrest (ICD-10-CM = I46). Only adult patients aged 18 years or older at the time of OHCA were included. In addition, to focus on non-traumatic OHCA, patients with traumatic cardiac arrest were excluded by limiting the cohort to those with ICD-10-CM diagnosis code I46. To define patients with return of spontaneous circulation (ROSC), this study excluded cases of individuals who died on the same day. The exposure group was defined as those diagnosed with COVID-19 (Supplementary Table 1) on the same day as the OHCA. The comparison group consisted of individuals who had never been diagnosed with COVID-19 from six months before the OHCA date to after the OHCA date. The index date was the date of the OHCA. The primary outcome of interest in this study was to estimate the risk of mortality between the COVID-19 and non-COVID-19 groups with a 3-month follow-up period.

Baseline characteristics were obtained from records spanning one year prior to the index date up until one day before the index date. Demographic variables of interest included age, sex, race, and body mass index (BMI). Relevant baseline comorbidities included hypertensive diseases, disorders of lipoprotein metabolism and other lipidemias, ischemic heart diseases, chronic kidney disease, overweight and obesity, cerebrovascular diseases, liver diseases, chronic obstructive pulmonary disease, and malignant neoplasms. The related codes are listed in Supplementary Table 2. Medical utilization data encompassed ambulatory, emergency, and inpatient encounters.

### Ethics statement

This research received approval from the Institutional Review Board for Ethics at Chung Shan Medical University Hospital (IRB number: CS2-23180; Date of approval: Dec 15, 2023) and was conducted in alignment with the Declaration of Helsinki. Since the datasets used in this study contain de-identified information from the participants, the requirement for informed consent was waived.

### Statistical analysis

The balance of baseline characteristics between the matched cohorts was evaluated using standardized mean differences (SMD). Variables with an SMD below 0.1 were deemed well-matched. Kaplan-Meier survival curves and Cox proportional hazards models were employed to compare mortality risk between the two groups, with hazard ratios (HRs) and 95% confidence intervals (CIs) computed. Stratified analyses were carried out to assess the association between COVID-19 and mortality in subgroups defined by age, sex, antiviral treatment, COVID-19 variant epidemic periods (Alpha: January 1, 2020 - June 30, 2021; Delta: July 1, 2021 - December 31, 2021; Omicron: January 1, 2022 onwards), and COVID-19 vaccination (Supplementary Table 1), as well as to further investigate this association in the non-traumatic OHCA population (excluding those with trauma diagnoses coded as ICD-10-CM S00–S99). Antiviral treatments included the use of paxlovid, molnupiravir, and remdesivir within five days of a COVID-19 diagnosis. All analyses were completed using the built-in analysis interface of the TriNetX online platform.

## Results

### Baseline characteristics of the study cohorts

Our study included 2,781 patients who received a diagnosis of COVID-19 on the same day of OHCA (out-of-hospital cardiac arrest) and a control group of 18,475 patients who never received a COVID-19 diagnosis within the 6-month period encompassing the date of OHCA (both before and after the event). This study period spanned OHCA events occurring between January 1, 2020, to December 31, 2023.This selection is depicted in Fig. 1. Table 1 lists the distribution of baseline demographics, BMI categories, medical utility and comorbidities between the COVID-19 cohort and the non–COVID-19 cohort before and after propensity score matching (PSM).

**Table 1:** Demographic characteristics of COVID-19 and Non COVID-19

	Before PSM			After PSM		
	COVID-19 *N* = 2781	Non-COVID-19 *N* = 18,475	SMD	COVID-19 *N* = 2776	Non-COVID-19 *N* = 2776	SMD
**Age**	63.17 ± 16.18	62.05 ± 17.91	0.066	63.16 ± 16.19	63.63 ± 16.76	0.028
**Sex**						
Female	1034 (37.18)	6915 (37.43)	0.005	1032 (37.18)	1028 (37.03)	0.003
Male	1736 (62.42)	11,368 (61.53)	0.018	1733 (62.43)	1737 (62.57)	0.003
Unknown Gender	11 (0.40)	192 (1.04)	0.076	11 (0.40)	11 (0.40)	< 0.001
**Race**						
White	1636 (58.83)	11,222 (60.74)	0.039	1633 (58.83)	1639 (59.04)	0.004
Black or African American	577 (20.75)	3694 (20.00)	0.019	577 (20.79)	578 (20.82)	0.001
Asian	165 (5.93)	1100 (5.95)	0.001	165 (5.94)	189 (6.81)	0.035
American Indian or Alaska Native	15 (0.54)	59 (0.32)	0.034	14 (0.50)	14 (0.50)	< 0.001
Native Hawaiian or Other Pacific Islander	62 (2.23)	414 (2.24)	0.001	62 (2.23)	56 (2.02)	0.015
Other Race	149 (5.36)	683 (3.70)	0.080	148 (5.33)	130 (4.68)	0.030
Unknown Race	177 (6.37)	1303 (7.05)	0.028	177 (6.38)	170 (6.12)	0.010
**BMI**	30.39 ± 8.71	28.54 ± 8.08	0.221	30.40 ± 8.71	28.95 ± 7.90	0.175
**Medical utility**						
Ambulatory	1455 (52.32)	10,070 (54.51)	0.044	1452 (52.31)	1433 (51.62)	0.014
Emergency	1125 (40.45)	7281 (39.41)	0.021	1122 (40.42)	996 (35.88)	0.094
Inpatient Encounter	840 (30.21)	5890 (31.88)	0.036	838 (30.19)	764 (27.52)	0.059
**Comorbidities**						
Hypertensive diseases	1173 (42.18)	7836 (42.41)	0.005	1171 (42.18)	1097 (39.52)	0.054
Disorders of lipoprotein metabolism and other lipidemias	862 (31.00)	5761 (31.18)	0.004	861 (31.02)	809 (29.14)	0.041
Ischemic heart diseases	611 (21.97)	4292 (23.23)	0.030	611 (22.01)	576 (20.75)	0.031
Chronic kidney disease	614 (22.08)	3814 (20.64)	0.035	614 (22.12)	572 (20.61)	0.037
Overweight and obesity	383 (13.77)	2106 (11.40)	0.072	381 (13.73)	342 (12.32)	0.042
Cerebrovascular diseases	216 (7.77)	1723 (9.33)	0.056	216 (7.78)	217 (7.82)	0.001
Diseases of liver	187 (6.72)	1427 (7.72)	0.039	187 (6.74)	177 (6.38)	0.015
Chronic obstructive pulmonary disease	294 (10.57)	2339 (12.66)	0.065	294 (10.59)	273 (9.83)	0.025
Malignant neoplasms of lip, oral cavity and pharynx	11 (0.40)	116 (0.63)	0.033	11 (0.40)	12 (0.43)	0.006
Malignant neoplasms of digestive organs	28 (1.01)	378 (2.05)	0.085	28 (1.01)	33 (1.19)	0.017
Malignant neoplasms of respiratory and intrathoracic organs	42 (1.51)	363 (1.97)	0.035	42 (1.51)	43 (1.55)	0.003
Malignant neoplasms of bone and articular cartilage	0 (0.00)	21 (0.11)	0.048	0 (0.00)	10 (0.36)	0.085
Melanoma and other malignant neoplasms of skin	25 (0.90)	196 (1.06)	0.016	25 (0.90)	36 (1.30)	0.038
Malignant neoplasms of mesothelial and soft tissue	10 (0.36)	67 (0.36)	0.001	10 (0.36)	10 (0.36)	< 0.001
Malignant neoplasms of breast	20 (0.72)	188 (1.02)	0.032	20 (0.72)	15 (0.54)	0.023
Malignant neoplasms of female genital organs	12 (0.43)	101 (0.55)	0.017	12 (0.43)	10 (0.36)	0.011
Malignant neoplasms of male genital organs	34 (1.22)	295 (1.60)	0.032	34 (1.23)	44 (1.59)	0.031
Malignant neoplasms of urinary tract	27 (0.97)	166 (0.90)	0.008	27 (0.97)	20 (0.72)	0.028
Malignant neoplasms of eye, brain and other parts of central nervous system	0 (0.00)	44 (0.24)	0.069	0 (0.00)	0 (0.00)	< 0.001
Malignant neoplasms of thyroid and other endocrine glands	10 (0.36)	43 (0.23)	0.023	10 (0.36)	10 (0.36)	< 0.001
Malignant neoplasms of ill-defined, other secondary and unspecified sites	65 (2.34)	707 (3.83)	0.086	65 (2.34)	53 (1.91)	0.030
Malignant neuroendocrine tumors	10 (0.36)	33 (0.18)	0.035	10 (0.36)	10 (0.36)	< 0.001
Secondary neuroendocrine tumors	0 (0.00)	13 (0.07)	0.038	0 (0.00)	10 (0.36)	0.085
Malignant neoplasms of lymphoid, hematopoietic and related tissue	76 (2.73)	338 (1.83)	0.061	73 (2.63)	61 (2.20)	0.028

Before propensity score matching, the average age in the COVID-19 cohort was 63.17 ± 16.18 years compared to 62.05 ± 17.91 years in the non- COVID-19 cohort, that was less than small effect size. The proportion of female sex was 37.18% in the COVID-19 cohort versus 37.43% in the non- COVID-19 cohort, the difference falling under the small effect size. The proportion of White individuals in the COVID cohort (58.83%) was slightly lower compared to the non-COVID cohort (60.74%). Similarly, the proportion of Black or African American individuals was slightly higher in the COVID cohort (20.75%) compared to the non-COVID cohort (20%). These differences, however, along with those observed for other racial groups such as Asian, American Indian or Alaska Native, Native Hawaiian or Other Pacific Islander, and Other Race, all represent less than small effect sizes in both cohorts. Among those with recorded BMI, the average for the COVID-19 cohort was higher than the non-COVID-19 cohort, at 30.39 ± 8.71 versus 28.54 ± 8.08, respectively. The COVID-19 cohort also exhibited similar proportions of individuals utilizing various healthcare services compared to the non-COVID-19 cohort. These services included ambulatory care, emergency department visits, and inpatient hospital admissions. For baseline comorbidities, the proportion of individuals with various conditions in the COVID-19 cohort (e.g., hypertensive diseases, disorders of lipoprotein metabolism and other lipidemias, ischemic heart diseases, chronic kidney disease, overweight and obesity, cerebrovascular diseases, liver diseases, chronic obstructive pulmonary disease, and malignant neoplasms) showed only less than small effect size differences compared to the non-COVID-19 cohort.

After implementing propensity score matching, our study aligned 2,776 patients in each cohort. Post-matching, the distribution of demographics, medical utility and comorbidities were comparable (std-diff. <0.1) between the COVID cohort and non-COVID cohort.

### Mortality rates across varying follow-up durations

**Fig. 2:**
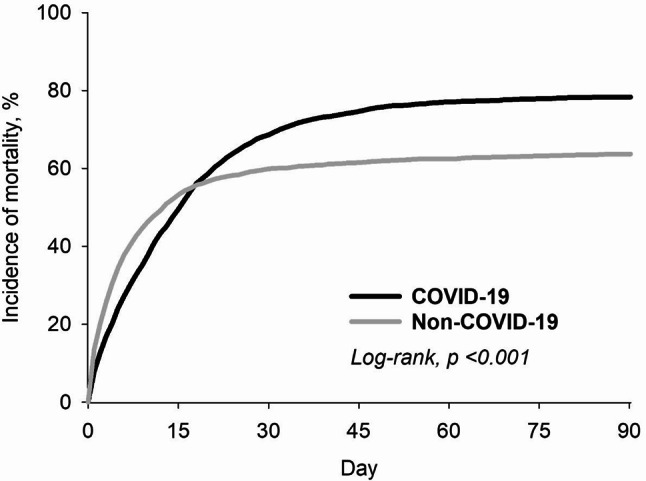
Kaplan–Meier analysis for mortality between COVID-19 and non-COVID-19 patients with a 3-month follow-up period

In the Kaplan-Meier analysis (Fig. 2) comparing mortality between COVID-19 and non-COVID-19 patients, the incidence of mortality was significantly higher in the COVID-19 group starting at 15 days after the OHCA event (*p* < 0.01). Table 2 displays the results of the Cox regression analysis for the risk of mortality and in COVID-19 cohort compared with non-COVID-19 cohort after PSM. The mortality rate of the COVID-19 cohort was lower than that of the non-COVID-19 cohort at 14 days follow-up (HR 0.82, 95% CI: 0.76–0.88), but higher at 90 days follow-up (HR 1.16, 95% CI: 1.09–1.24).

**Table 2:** Risk of mortality among different follow-up period

	COVID-19		Non-COVID-19		
	*N*	No. of event	*N*	No. of event	HR (95% C.I.)
Follow-up period					
14 days	2776	1263	2776	1352	0.82 (0.76–0.88)
30 days	2776	1849	2776	1555	1.09 (1.02–1.17)
90 days	2776	2081	2776	1666	1.16 (1.09–1.24)
Exclude death within 14 days					
15–30 days	1250	469	1222	166	2.93 (2.46–3.50)
15–90 days	1250	668	1222	235	3.22 (2.78–3.74)
Exclude death within 5 days					
6–30 days	2008	1100	1724	598	1.67 (1.51–1.84)
6–90 days	2008	1383	1724	709	1.87 (1.71–2.05)

Further analysis revealed that the COVID-19 cohort consistently exhibited a higher mortality rate compared to the non-COVID-19 cohort, even after excluding early deaths (death within 14 days or death within 5 days). We observed a similar trend of higher mortality in the COVID-19 group compared to the non-COVID-19 group across both the shorter (15–30 days and 6–30 days) and longer follow-up periods (15–90 days and 6–90 days).

### Subgroup analyses by age, sex, antivirals, and COVID-19 virus variant epidemic period

**Fig. 3:**
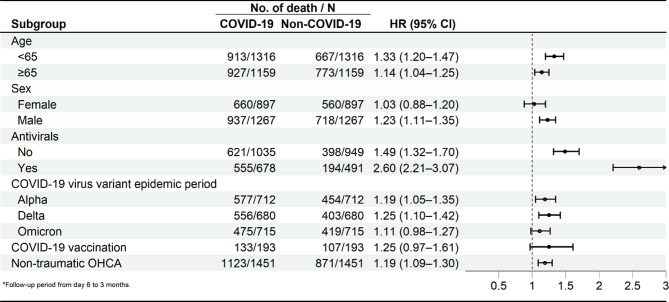
Forest plot of the risk of mortality among stratifications by age, sex, antivirals, and COVID-19 virus variant epidemic period with a 3-month follow-up period

**Fig. 4:**
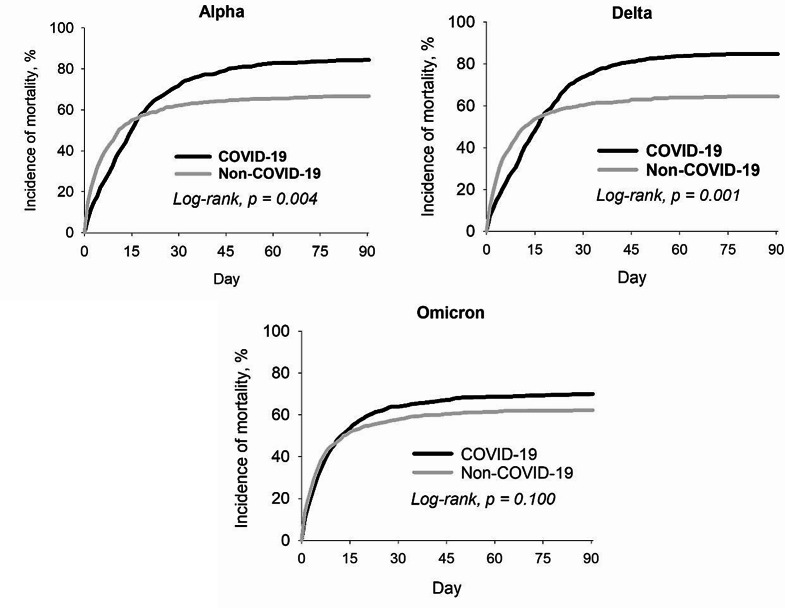
Risk of mortality among different COIVD-19 virus variant epidemic period with a 3-month follow-up period

The results of subgroup analyzed for the risk of mortality were detailed in Fig. 3. When stratified by age, the hazard ratio (HR) with 95% confidence intervals (CI) was 1.33 (95% CI:1.20–1.47) for patients aged < 65, 1.14 (95% CI:1.04–1.25) for those aged ≥ 65. Male patients who diagnosed COVID-19 exhibited a higher increased risk of mortality (HR: 1.23, 95% CI: 1.11–1.35). However, the analysis revealed no significant difference in mortality between female patients with and without COVID-19. The increased mortality risk associated with COVID-19 infection remained evident regardless of whether patients received specific COVID-19 treatments and among the non-traumatic OHCA population. Our study revealed a significant disparity in mortality rates between COVID-19 patients and non-COVID-19 patients, specifically during the SARS-CoV-2 Alpha and Delta variant epidemic period (HR: 1.19, 95% CI: 1.05–1.35; HR: 1.25, 95% CI: 1.10–1.42). However, this difference was not observed during the Omicron variant epidemic periods. In the stratified analysis based on COVID-19 vaccination status, no significantly higher mortality risk was observed among COVID-19 patients either (Figs. 3 and 4).

## Discussion

Using data from the TriNetX analysis platform during the COVID-19 pandemic for investigation the effect of COVID-19 infection in OHCA patients, we observed an opposite trend in mortality rates among OHCA patients between COVID-19 and non-COVID-19 cohort with different follow up period. The mortality rate for COVID-19 patients was lower in the shorter follow-up period (14 days) compared to non-COVID-19 patients (HR: 0.82, 95% CI: 0.76–0.88). However, this trend reversed when the observation period was extended (90 days). To our knowledge, published studies up to now focus on comparing OHCA incidence, characteristics and mortality during the pandemic to pre-pandemic periods and identifying the unique features of OHCA patients during COVID-19 pandemic. However, a notable gap exists in the literature regarding the in-depth analysis of outcomes and mortality rates specifically among confirmed COVID-19 infection among OHCA patients. Our study revealed a striking disparity in mortality rates between COVID-19-positive and COVID-19-negative OHCA patients. Notably, COVID-19-positive patients exhibited a lower mortality rate within the first 14 days of hospitalization compared to their COVID-19-negative counterparts. However, this trend reversed thereafter, with COVID-19-negative patients experiencing a lower mortality rate beyond the two-week mark. This observed pattern could be attributed to several underlying factors. One likely reason is the uneven allocation of healthcare resources during the pandemic. Ethical discussions have pointed out that prioritizing COVID-19-related care may unintentionally reduce the availability of resources for non-COVID-19 patients, raising concerns about fairness in medical care allocation [10]. Evidence also supports this concern; a recent study analyzing mortality data in the United States found that deaths from non-COVID-19 causes increased during the pandemic, likely due to the reallocation of medical resources and decreased access to routine care [11]. To reduce the impact of such service interruptions and their possible effects on population health, it is important to establish clear policies or guidelines that help maintain access to care for non-COVID-19 patients during pandemics and other health emergencies. This approach may help prevent the increased mortality burden seen in non-COVID-19 patients in the later stages of hospitalization.

Past studies have consistently reported an increase in the incidence and mortality of OHCA patients during the pandemic [1, 7]. Additionally, they documented a decrease in the rate of shockable rhythm as the initial presenting rhythm, as well as a decrease in the rates of ROSC, survival to hospital admission, and survival to hospital discharge [12]. The pandemic likely drives the increase in OHCA deaths through both direct and indirect mechanisms. COVID-19 infection can directly precipitate OHCA through several mechanisms [13]. These include severe respiratory failure leading to hypoxia, venous thromboembolism, and cardiac inflammation manifested as myocarditis, acute coronary thrombosis, and arrhythmias [14]. In addition, the pandemic has indirectly impacted healthcare utilization, leading to decreased patient visits, delayed care, and limited access to medical services [15]. It has also contributed to reduced physical activity and increased social isolation [16]. Pandemic-related limitations on accessing healthcare professionals at established chronic disease clinics may have contributed to treatment postponement and potential progression of untreated illnesses to a critical stage [17]. The COVID-19 pandemic has also significantly impacted emergency medical services (EMS) response times for OHCA patients. Studies have shown that EMS response times were prolonged during the pandemic compared to pre-pandemic periods. This delay can be attributed to effect caused by factors like increased information gathering. Dispatchers needed more time to collect details about potential COVID-19 symptoms, travel history, and contact with high-risk individuals. Additionally, personal protective equipment (PPE) preparation played a role. Emergency medical technicians (EMTs) were required to don PPE before entering the scene, adding to the overall response time [18]. Furthermore, as the COVID-19 situation continued to evolve, the reallocation of medical resources and manpower, the establishment of management protocols, and the development of specific patient care pathways were all constantly changing [19]. The allocation of hospital resources (beds, equipment, staff, personal protective equipment, consumables, oxygen, medication, mortuary) between COVID-19 and non-COVID-19 patients posed a significant challenge during the COVID-19 pandemic [20]. The diversion of substantial resources to care for COVID-19 patients compelled the suspension of routine healthcare services, potentially causing severe delays in hospitalization for non-COVID-19 patients [21]. The ability of emergency healthcare services (both pre-hospital and in-hospital) to deliver high-quality care may have been also compromised due to immense resource strain.

COVID-19 infection, including long-COVID, raises the risk of both short-term and long-term cardiovascular complications and all-cause mortality [22]. The past literature on COVID-19 employed a variety of timeframes to define the short- and long-term phase of the illness. These definitions ranged from 21 days after the COVID infection date (index date) for the acute phase to 60 days, 3 months, and even a year [22–24]. Past studies also have described the outcomes of individuals with COVID-19, both in the early stages of infection and during hospitalization. A study by Yovita et al. found that mortality rates tend to increase after the second week of hospitalization, and this may be associated with secondary bacterial infections [9]. Data from another study further corroborated the notion that SARS-CoV-2 infection enhanced susceptibility and pathogenicity to bacterial coinfection. Notably, the study also demonstrated that bacterial coinfection instigated at 5 or 7 days post-viral infection (pvi) exacerbated mortality rates, while coinfection initiated at 3 d pvi did not exhibit a similar impact [25]. Given that a significant proportion (approximately 50%) of COVID-19 deaths have been associated with secondary bacterial infections, according to existing reports [26, 27]. Furthermore, secondarily infected patients had longer hospital stay, higher odds of ICU admission, mortality, and invasive procedures [27]. While various factors contribute to the overall higher mortality rate among OHCA patients during the COVID-19 pandemic, a refined follow-up period analysis in our study suggested that the pandemic’s prioritized and comprehensive medical care for COVID-19 positive individuals might have led to a lower mortality rate within a very short timeframe (14 days post-OHCA) for COVID-19 diagnosed patients. However, extending the follow-up period revealed a higher mortality rate associated with COVID-19 infection due to the potential for subsequent infections and complications.

There are also other factors associated with a higher risk of severe illness and death from COVID-19 in infected individuals. These include older age, being male, having underlying health conditions, and racial or ethnic disparities [28]. Besides, previous study found that antiviral medication was associated with significantly reduced inpatient mortality among patients hospitalized with COVID-19 [29]. Moreover, the previous article emphasized the significance of examining outcomes for hospitalized COVID-19 patients throughout the pandemic to understand the influence of various SARS-CoV-2 variants. It compared the 28-day in-hospital mortality rates for patients infected with the Wild-type, Alpha, Delta, and Omicron variants. The findings corroborated the notion that in-hospital mortality rates have indeed declined over the course of the pandemic, particularly since the emergence of the Omicron variant. While differences in virulence among SARS-CoV-2 variants may have also played a role, the observed decrease in-hospital mortality in this study appeared to be a combined effect of immunity from vaccinations and prior infections [30]. Given the multifaceted nature of mortality rates, our study employed subgroup analyses to investigate the impact of specific factors on outcomes. Furthermore, given the potential role of secondary bacterial infections or other complications in the higher mortality rates observed beyond the second week of hospitalization, it was crucial to implement effective measures for identifying, preventing, and managing these complications in both COVID-19-positive and COVID-19-negative OHCA patients.

### Study strengths and limitations

This study possessed several strengths that contribute to the reliability of its evidence. First, it utilized a large sample size and included a geographically diverse patient population. This surpassed previous research, which often relied on data from single healthcare systems in specific locations with limited patient numbers. Additionally, we employed a well-defined and accurate follow-up period to ensure consistent data collection. Furthermore, we conducted subgroup analyses stratified by factors such as gender, age, antiviral medication use, and specific SARS-CoV-2 variants. This allowed us to assess the impact of these variables on the study’s accuracy in patients with OHCA. To minimize misclassification bias, we strictly restricted COVID-19 diagnoses to individuals confirmed positive via RNA or antigen tests. However, our study acknowledged several limitations. Firstly, OHCA patient mortality was variable and depended on a confluence of factors, including patient characteristics, emergency medical services (EMS) response, and hospital resuscitation capabilities. Additionally, due to limitations in the TriNetX database, we could not access detailed prehospital information such as bystander CPR, response time, or defibrillation timing. As a result, important factors in the chain of survival could not be incorporated into our propensity score matching, and this remains a limitation of our study. Moreover, in-hospital developments, such as the presence and type of secondary infections, could influence outcomes. While we aimed to adjust for most confounding variables between the study and control groups, some degree of misclassification bias and residual confounding remains inevitable. This may be due to unmeasured comorbidities, baseline severity of existing conditions, or lifestyle factors. Furthermore, due to the limitations of the TrinetX platform, we were unable to align the index dates between the two groups, which led to a potential risk of selection bias caused by time-based sampling. To ensure the reliability of our study, we further analyzed different COVID-19 variant epidemic periods. Finally, specific neurological outcomes such as Cerebral Performance Category (CPC) scores were not available in the TriNetX database, limiting our ability to assess neurological function post-resuscitation. As such, our analysis was restricted to mortality, which was the most consistently reported and comparable outcome across the cohort. Future studies should aim to incorporate neurological outcome measures to more comprehensively evaluate the impact of OHCA in different patient populations.

## Conclusions

Our study highlights a significant disparity in mortality rates between COVID-19-positive and COVID-19-negative OHCA patients, with a notable shift in trends depending on the follow-up period. Initially, COVID-19-positive patients exhibited lower mortality rates within the first 14 days of hospitalization. However, over the long term, COVID-19 was associated with a higher risk of mortality in OHCA patients.

## Electronic supplementary material

Below is the link to the electronic supplementary material.

Supplementary Material 1

## Data Availability

Research data supporting this publication can be accessed through the TriNetX platform, available at https://trinetx.com/. Data sharing is not applicable to this article, as no datasets were generated or analyzed during the current study.

## Abbreviations

BMI: Body mass index
CPR: Cardiopulmonary resuscitation
EHRs: Electronic health records
EMS: Emergency medical services
OHCA: Out-of-Hospital Cardiac Arrest
PSM: Propensity score matching
ROSC: Return of spontaneous circulation
