# Supplementary material for: Impact of COVID-19 on mortality in out-of-hospital cardiac arrest patients with return of spontaneous circulation: a retrospective cohort study
Source: Scand J Trauma Resusc Emerg Med. 2025 May 1;33:75. doi: 10.1186/s13049-025-01395-2 (PMC12044919; doi:10.1186/s13049-025-01395-2)
Supplement: Supplementary file 1 — Supplementary Material 1 [file 13049_2025_1395_MOESM1_ESM.pdf]

Supplementary Table 1 Codes used for COVID-19 and SARS-CoV-2 (COVID-19) Vaccine

Code	Content
Lab: 9088	SARS coronavirus 2 and related RNA [Presence] (labResult: Positive)
Lab: 9089	SARS coronavirus 2 IgG IgM Ab [Presence] in Serum or Plasma (labResult: Positive)
LOINC: 94505-5	SARS-CoV-2 (COVID-19) IgG Ab [Units/volume] in Serum or Plasma by Immunoassay (at least 0.10 [arb'U]/mL)
LOINC: 94506-3	SARS-CoV-2 (COVID-19) IgM Ab [Units/volume] in Serum or Plasma by Immunoassay (at least 0.10 [arb'U]/mL)
LOINC: 94562-6	SARS-CoV-2 (COVID-19) IgA Ab [Presence] in Serum or Plasma by Immunoassay (labResult: Positive)
LOINC: 94762-2	SARS-CoV-2 (COVID-19) Ab [Presence] in Serum or Plasma by Immunoassay (labResult: Positive)
LOINC: 94769-7	SARS-CoV-2 (COVID-19) Ab [Units/volume] in Serum or Plasma by Immunoassay (at least 0.10 [IU]/mL)
LOINC: 94558-4	SARS-CoV-2 (COVID-19) Ag [Presence] in Respiratory specimen by Rapid immunoassay (labResult: Positive)
LOINC: 95209-3	SARS-CoV+SARS-CoV-2 (COVID-19) Ag [Presence] in Respiratory specimen by Rapid immunoassay (labResult: Positive)
LOINC: 96119-3	SARS-CoV-2 (COVID-19) Ag [Presence] in Upper respiratory specimen by Immunoassay (labResult: Positive)
ICD-10-CM: U07.1	COVID-19
**COVID-19 Vaccination**	
CVX: 213	SARS-CoV-2 (COVID-19) Vaccine
CPT: 91300	Severe acute respiratory syndrome coronavirus 2 (SARS-CoV-2) (coronavirus disease [COVID-19]) vaccine, mRNA-LNP, spike protein, preservative free, 30 mcg/0.3 mL dosage, diluent reconstituted, for intramuscular use (deprecated 2024)
CPT: 91301	Severe acute respiratory syndrome coronavirus 2 (SARS-CoV-2) (coronavirus disease [COVID-19]) vaccine, mRNA-LNP, spike protein, preservative free, 100 mcg/0.5 mL dosage, for intramuscular use (deprecated 2024)
CPT: 91302	Severe acute respiratory syndrome coronavirus 2 (SARS-CoV-2) (coronavirus disease [COVID-19]) vaccine, DNA, spike protein, chimpanzee adenovirus Oxford 1 (ChAdOx1) vector, preservative free, 5x1010 viral particles/0.5 mL dosage, for intramuscular use (deprecated 2024)
CPT: 91303	Severe acute respiratory syndrome coronavirus 2 (SARS-CoV-2) (coronavirus disease [COVID-19]) vaccine, DNA, spike protein, adenovirus type 26 (Ad26) vector, preservative free, 5x1010 viral particles/0.5 mL dosage, for intramuscular use (deprecated 2024)
CPT: 91305	Severe acute respiratory syndrome coronavirus 2 (SARS-CoV-2) (coronavirus disease [COVID-19]) vaccine, mRNA-LNP, spike protein, preservative free, 30 mcg/0.3 mL dosage, tris-sucrose formulation, for intramuscular use (deprecated 2024)
CPT: 91306	Severe acute respiratory syndrome coronavirus 2 (SARS-CoV-2) (coronavirus disease [COVID-19]) vaccine, mRNA-LNP, spike protein, preservative free, 50 mcg/0.25 mL dosage, for intramuscular use (deprecated 2024)
CPT: 91307	Severe acute respiratory syndrome coronavirus 2 (SARS-CoV-2) (coronavirus disease [COVID-19]) vaccine, mRNA-LNP, spike protein, preservative free,

	10 mcg/0.2 mL dosage, diluent reconstituted, tris-sucrose formulation, for intramuscular use (deprecated 2024)
CPT: 91308	Severe acute respiratory syndrome coronavirus 2 (SARS-CoV-2) (coronavirus disease [COVID-19]) vaccine, mRNA-LNP, spike protein, preservative free, 3 mcg/0.2 mL dosage, diluent reconstituted, tris-sucrose formulation, for intramuscular use (deprecated 2024)
CPT: 91309	Severe acute respiratory syndrome coronavirus 2 (SARS-CoV-2) (coronavirus disease [COVID-19]) vaccine, mRNA-LNP, spike protein, preservative free, 50 mcg/0.5 mL dosage, for intramuscular use (deprecated 2024)
CPT: 91322	Severe acute respiratory syndrome coronavirus 2 (SARS-CoV-2) (coronavirus disease [COVID-19]) vaccine, mRNA-LNP, 50 mcg/0.5 mL dosage, for intramuscular use

Abbreviations: [arb'U]/mL, arbitrary unit.
LOINC, Logical Observation Identifiers Names and Codes.
ICD-10-CM: International Classification of Diseases, Tenth Revision, Clinical Modification.
CVX: Vaccine administered code.
CPT: Current Procedural Terminology.

Supplementary Table 2 Codes used for comorbidities

ICD-10-CM	Content
I10-I1A	Hypertensive diseases
E78	Disorders of lipoprotein metabolism and other lipidemias
I20-I25	Ischemic heart diseases
N18	Chronic kidney disease
E66	Overweight and obesity
I60-I69	Cerebrovascular diseases
K70-K77	Diseases of liver
J44	Chronic obstructive pulmonary disease
C00-C14	Malignant neoplasms of lip, oral cavity and pharynx
C15-C26	Malignant neoplasms of digestive organs
C30-C39	Malignant neoplasms of respiratory and intrathoracic organs
C40-C41	Malignant neoplasms of bone and articular cartilage
C43-C44	Melanoma and other malignant neoplasms of skin
C45-C49	Malignant neoplasms of mesothelial and soft tissue
C50-C50	Malignant neoplasms of breast
C51-C58	Malignant neoplasms of female genital organs
C60-C63	Malignant neoplasms of male genital organs
C64-C68	Malignant neoplasms of urinary tract
C69-C72	Malignant neoplasms of eye, brain and other parts of central nervous system
C73-C75	Malignant neoplasms of thyroid and other endocrine glands
C76-C80	Malignant neoplasms of ill-defined, other secondary and unspecified sites
C7A-C7A	Malignant neuroendocrine tumors
C7B-C7B	Secondary neuroendocrine tumors
C81-C96	Malignant neoplasms of lymphoid, hematopoietic and related tissue
